# Carpal tunnel syndrome caused by cysticercosis

**DOI:** 10.4103/0970-0358.73454

**Published:** 2010

**Authors:** S. R. Sharma, Nalini Sharma, M. E. Yeolekar

**Affiliations:** North Eastern Indira Gandhi Regional Institute of Medical Sciences, Shillong, India

**Keywords:** Carpal tunnel syndrome, cysticercosis, entrapment neuropathy, median nerve

## Abstract

We present a case of carpal tunnel syndrome (CTS) due to compression of the median nerve within the carpal tunnel, caused by cysticercosis. Nerve conduction studies revealed severe CTS. Magnetic resonance imaging suggested an inflammatory mass compressing the median nerve in carpal tunnel. The histological diagnosis was consistent with cysticercosis. The case resolved with conservative treatment. Such solitary presentation of entrapment median neuropathy as CTS caused by cysticercosis is extremely rare. To our knowledge, this is the only case of its kind reported in literature till date.

## INTRODUCTION

Carpal tunnel syndrome (CTS) is a constellation of signs and symptoms resulting from compression of the median nerve in the carpal tunnel.[1] CTS is the most commonly encountered entrapment neuropathy with an incidence of 139 per 100,000 person-years for men and 506 per 100,000 person-years for women.[2] The classic symptoms of CTS are numbness and paraesthesia in the first three fingers of the hand, which is commonly exacerbated at night.[3] The diagnostic signs include sensory loss along the lateral aspect of the hand, motor weakness and wasting of abductor pollicis brevis (APB) muscle and eliciting Tinel’s and Phalen’s sign at the wrist.

The nerve conduction study (NCS) study is a definite diagnostic test for CTS, with a high degree of sensitivity and specificity(4). *Taenia solium*, the pork tapeworm is endemic in Mexico, Central and South America, Africa, India, Pakistan and China. Human cysticercosis is caused by dissemination of embryos of *T. solium* from the intestine via the hepatoportal system to the tissues and organs of the body.[5] Apart from the involvement of the central nervous system, subcutaneous tissue and muscle by cysticercosis, less frequently, cysticerci may localise in other organs like eyes, tongue, oral cavity, breast, heart and lungs.[6–8]

We present a case of CTS due to compression of the median nerve within the carpal tunnel, caused by cysticercosis.

## CASE REPORT

**Figure 1:**
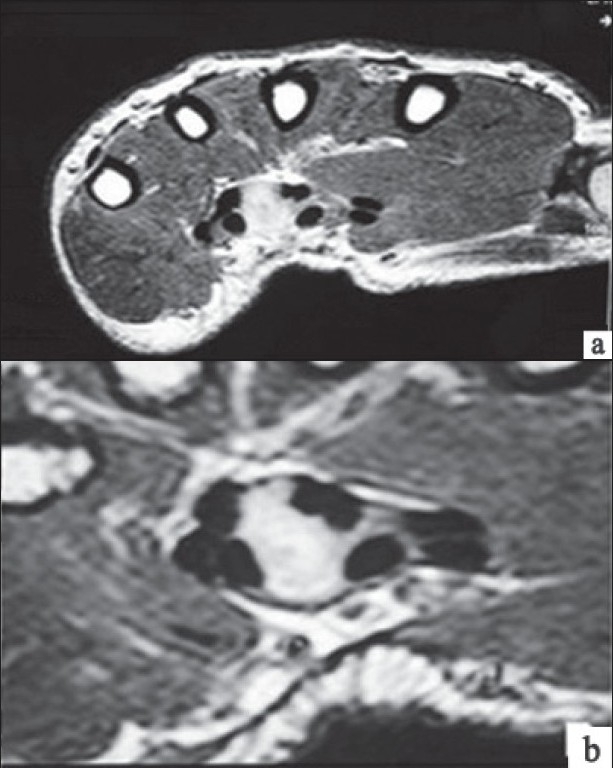
MRI wrist joint showing compression of median nerve by cysticercosis

A 38-year-old man presented with progressively worsening tingling, pins and needles in the radial four digits, loss of strength and awakening nocturnal pain in the right hand of 2 years duration. Physical examination showed swelling measuring 3–4 cm over volar aspect of the wrist. A mild thenar atrophy was observed and phalen’s test and tinel’s sign were positive. NCS of the median nerve revealed an absence of digit to wrist sensory Nerve Action Potential (NAP) on the right side and distal motor latency was 6.2 ms. Needle Electro Myo-graphy findings included fibrillation activity, decreased recruitment and abnormalities (large and long duration polyphasic Motor Unit Potentials (MUPs) in configuration of MUPs. The left hand was neurophysiologically intact. Both clinical symptoms and signs and neurophysiological tests [according to American Association of the Electro diagnostic Medicine (AAEM) criteria] showed severe CTS in right hand, while the left hand was completely healthy, implying a secondary disease. Magnetic resonance imaging (MRI) [Figure 1] with contrast revealed a sharply defined elliptical mass enhancing with contrast, in the deep palmar space extending into the carpal tunnel and compressing the median nerve. Fine needle aspiration cytology revealed a chronic inflammatory mass infiltrated with predominantly macrophages and lymphocytes and parasitic fragments, consistent with cysticerosis. Cysticercus serology was positive. He was started on a short course of steroids for 2 weeks, along with nonsteroidal anti-inflammatory drugs. The hand was put on a splint and physiotherapy was started. There was remarkable improvement in symptoms with reduction of swelling size. Although surgical excision was initially planned, since he improved by conservative management alone, it was abandoned. He became asymptomatic and his deformity completely corrected in 2.5 months of follow-up.

## DISCUSSION

It is widely known that idiopathic CTS usually presents with bilateral symptoms.[9–11] In patients who present with unilateral symptoms, 38–50% were reported to have positive electro diagnostic test results in the asymptomatic, contralateral hand.[12] CTS is the most common entrapment neuropathy, with a prevalence of 10–20% for symptoms in the population-based studies.[13] Space-occupying lesions are known to cause CTS and the incidence of space-occupying lesions in unilateral CTS is higher than that in bilateral CTS.[14] Cysticercosis is a systematic illness caused by dissemination of the larval forms of the pork tapeworm, *T. solium*. Man is the definitive host for *T. solium*, and humans acquire this disease by ingesting the eggs of *T. solium* from food or water contaminated by human faeces or autoinfection. The larvae enter the bloodstream, migrate, and encyst in tissue, usually striated muscle or brain. Less frequently, cysticerci may localise in other organs. The encysted larvae may remain asymptomatic or may provoke granulomatous inflammatory response depending on the anatomical site.[5]

The presenting muscle symptoms of cysticercosis vary greatly. Unlike ceberal disease, muscle cysticercosis is not grave but can cause morbidity of varying severity.[15,16] Most of the patients are asymptomatic, and characteristic elliptical, calcified lesions are detected incidentally on plain X-Ray films of the extremities. It can also present as acute myositis as a result of a host inflammatory response to dying larvae, as mass lesions, myopathy, or rarely as muscular pseudo hypertrophy, depending on the parasite burden.[15]

Apart from peripheral neuropathy, cranial nerve neuropathies caused by cysticerosis, involving the second, third, fourth and fifth cranial nerves, have been described in the literature.[17–18]

Investigations in entrapment neuropathy cases are directed to confirm the area of entrapment and to document the extent of the pathology. Cysticerci are rarely seen in plain radiographs as multiple fusiform, cigar shaped calcifications within the muscles or as multiple punctuate soft tissue calcifications. MRI is far superior to computed tomography in detecting and evaluating the stage of cysticercosis.[19] The radiographic appearance of cysticercosis correlates with its pathological features and reflects the stage of maturation of the disease. Initially, when the parasite is viable, a fluid-filled cyst without peripheral enhancement is observed. Later, as in our case, peripherally enhancing cystic lesions after gadolinium injection are observed and correlate with the inflammatory host-tissue response that occurs during leakage of fluid or during death of the parasites associated with varying amounts of oedema. The final radiographic appearance is that of an elliptical, non–fluid-filled, calcified lesion. The role of MRI is increasing in diagnosing entrapment neuropathies and other pathological conditions of the wrist. Definitive diagnosis requires histopathological demonstration of the cysticercus. A needle or an open biopsy is used in determining the aetiology of cutaneous and muscular nodules.[18] It should be emphasised, however, that the appearance of the parasite varies with the degree of cyst degeneration or the plane of sectioning. In our case, we found a dense accumulation of mononuclear cells probably representing vigorous granulomatous inflammatory response to dying larvae. Serological diagnosis of cysticercosis gives additional clues to the diagnosis as in our case.

In cases of entrapment neuropathy, conservative approach in the form of limb positioning using splints, physiotherapy and anti-inflammatory drugs is tried initially. Surgical intervention is indicated if increasing paraesthesia occurs despite adequate conservative treatment and at the first sign of motor changes. For symptomatic cysticercus cysts outside the central nervous system, the most favourable approach is surgical resection. Hence, in our case, although initially surgical approach was planned, surgical intervention was withheld as the case showed dramatic improvement with conservative management.

## CONCLUSION

In endemic countries, cysticercosis can have an extremely varied presentation and should be a part of differential diagnosis while evaluating CTS. Imaging studies, especially MRI, which are usually not used in idiopathic CTS should be remembered in patients with unilateral symptoms, especially with a long history and when the symptomatic hand shows severe neurophysiological impairment. Early identification of the cause of CTS is essential as early appropriate intervention helps to avoid surgery and promotes complete recovery.
